# Birth cohort, sex and educational disparities in the trajectories of body mass index in Taiwan: a longitudinal study

**DOI:** 10.1186/s12889-022-12762-4

**Published:** 2022-02-28

**Authors:** Tzu-Jung Wong, Tsung Yu, Ly-yun Chang, Xiang Qian Lao

**Affiliations:** 1 Department of Healthcare Information and Management, School of Health Technology, Ming Chuan University, Taoyuan, Taiwan; 2 Department of Academic Clinical Programme, National Dental Centre, Singapore, Singapore; 3 Department of Public Health, College of Medicine, National Cheng Kung University, 1 University Road, Tainan, Taiwan; 4 Institute of Sociology, Academia Sinica, Taipei, Taiwan; 5 Jockey Club School of Public Health and Primary Care, the Chinese University of Hong Kong, Hong Kong, SAR China

**Keywords:** Cohort effect, Education, Sex, Body mass index, Health disparities

## Abstract

**Background:**

Taiwan has gone through rapid industrialization, urbanization and economic growth in the 20th and early 21st centuries. Therefore, the population has experienced significant changes in the physical and social environment during the life course, which may affect the overall adiposity. Our aim was to examine the age trajectories of height, weight and body mass index (BMI) in the Taiwanese population and to explore the influences of sex, birth cohort and education.

**Methods:**

The sample comprised 572,358 residents between 20 and 94 years of age in Taiwan who attended at least one health examination during 1996 to 2017 in a cohort study. Repeated measures of body weight and height were collected using an auto-anthropometer. We conducted a series of linear mixed-effects growth curve models to examine the trajectory of height, weight, and BMI across the life course with stratification by sex.

**Results:**

Age-related trajectories of BMI differed between men and women and stronger cohort effects were observed among men, with younger cohorts having higher BMI. After holding cohort and age variables constant, men with junior high or lower education were shorter, thinner and had higher BMI than men with university or higher education (effect sizes: − 3.138 cm, *p* < 0.001; − 2.277 kg, *p* < 0.001; 0.121 kg/m^2^, *p* < 0.001, respectively). Women with junior high or lower education were shorter, heavier and had higher BMI than women with university or higher education (effect sizes: − 2.368 cm, *p* < 0.001; 2.417 kg, *p* < 0.001; 1.691 kg/m^2^, *p* < 0.001, respectively). The educational disparities in BMI were found to be larger among women.

**Conclusions:**

Our findings suggest that younger generations, especially men, and lower educational level individuals, particularly women, have increasing levels of BMI. The influence of age and cohort effects together with sex and educational disparities on adiposity should be highlighted when designing future interventions and policies regarding overweight and obesity.

## Background

Body mass index (BMI) is an easy anthropometric measure that captures general adiposity and correlates with various chronic diseases and mortality [1]. Worldwide, age-related trajectories of BMI have been studied in multiple cohorts [2–7]. Recently, Yang and colleagues combined four large cohort studies in the United States (US): National Longitudinal Study of Adolescent to Adult Health, Midlife in the US study, Americans’ Changing Lives Study, and the Health and Retirement Study, in which participants’ age ranged from 11 to 90 years of age and older [8]. They conducted pooled integrative data analysis and showed that the trajectory of BMI increases in the US population steadily from adolescence to middle age at 40–60 years and then decreases in later life, with variation in trajectories across different birth cohorts, sex, race and educational levels [8].

Since individuals from different birth cohorts may experience very diverse physical and social environments throughout their life course, most life course studies on the trajectories of BMI have found large birth cohort differences. A higher mean level of BMI and faster increase in BMI are often associated with more recent cohorts than earlier cohorts [9, 10]. The secular change of physical and social environment is obvious for most countries. Over time, people have been exposed to an environment that becomes more obesogenic, such as living a life style with a high-calorie diet and low physical activity [11, 12].

Besides, social disparities in the trajectory of BMI are also of research interest. Women, lower socioeconomic status and minor racial/ethnic groups are often found to have a higher prevalence of overweight and obesity [4, 13]. In their integrative data analysis, Yang et al. they showed that Hispanics and Blacks were associated with higher BMI than Whites [8]. When using parental and adulthood education as a marker for socioeconomic status, they found that a lower educational level was associated with higher BMI. Understanding the social disparities in BMI trajectories will help us identify their social determinants and design research and policies that target the most vulnerable groups.

Studies regarding the life course trajectories of BMI are scarce in Asian populations. Given that Taiwan has gone through rapid industrialization, urbanization and economic growth during the latter half of the twentieth century, there is a huge transition in the living environment and people’s health behaviors [14]. We would expect that each birth cohort was exposed to its unique physical and social environment during their life course and that thereby each cohort perhaps has its unique pattern of trajectory of height, weight, and BMI. Moreover, it is unclear whether there are disparities in the trajectory of height, weight, and BMI between men and women as well as among different educational levels. Our aim was to examine these questions, using a large longitudinal health examination database in Taiwan.

## Methods

### Study sample

The Taiwan MJ cohort is a dynamic longitudinal study of health examination; the cohort profile and related studies using data from this cohort have been published previously [15–17]. Briefly, since 1994 the MJ Health Management Institution, a private company, provided fee-for-service standard health examination programs for residents in Taiwan. Members of the programs paid for their own health examination or were sponsored by their employers to attend periodic health examinations. At each clinical visit, participants underwent standard health examinations including physical examination, blood tests and urine tests and completed questionnaires regarding the information regarding sociodemographics, medical history, health behaviors, and lifestyle. The cohort included participants who provided informed consent for the use of their data for research purposes. During 1996 to 2017, 615,353 participants joined the programs, yielding 1,448,034 medical examinations. The present study was approved by the ethics board at the National Cheng Kung University in Tainan, Taiwan (A-ER-108-081).

Our study sample included 597,924 participants aged 20–94 years who had at least one health examination from 1996 to 2017. Among them, 25,566 participants had missing information regarding their educational levels. We excluded these participants, resulting in a sample of 572,358 participants (273,879 men and 298,479 women). Each participant could have more than one visit to the MJ Health Management Institution; the number of visits ranged from one to 34. Most participants had only one (*n* = 311,306, 54.4%) or two (*n* = 104,934, 18.3%) health examination visits (data points) during 1996 to 2017.

### Measures

#### Dependent variables

The anthropometric data, including body weight and body height, were measured by auto-anthropometer in the MJ Health Management Institution (KN-5000A; Nakamura, Tokyo, Japan). Weight was measured without shoes and with participants wearing light indoor clothing and was recorded to the nearest 0.1 kg. Height was measured and recorded to the nearest 0.1 cm. BMI was calculated using weight (kg) divided by the square of height (m).

#### Independent variables

Participants were queried in the questionnaire about their highest educational level. There were seven categories: (1) Some elementary school (< 6 years), (2) Elementary school (6 years), (3) Junior high school (9 years), (4) Senior high school (12 years), (5) College (14 years), (6) University (16 years) and (7) Graduate school or higher (> 16 years). For simplicity, in the analysis we categorized educational levels into three: (1) Junior high school or lower (≤9 years), (2) Senior high school/college (10–15 years), and (3) University or higher (≥16 years). We defined nine categories of birth cohort: (1) before 1920, (2) 1920–1929, (3) 1930–1939, (4) 1940–1949, (5) 1950–1959, (6) 1960–1969, (7) 1970–1979 (8) 1980–1989, and (9) after 1990.

### Statistical analysis

To examine the association of age and birth cohort with height, weight, and BMI, we plotted the mean values of height, weight, and BMI against age in years with stratification by birth cohort and sex. We used linear mixed-effects growth curve models for data analysis to examine the trajectory of height, weight, and BMI across the life course. Linear mixed-effects growth curve models can deal with data that are unbalanced in time [18]; therefore, these models are particularly suitable for our data that were collected using a dynamic cohort study design where each participant had a different number of visits. To maximize data points in our analysis, we included all participants regardless of the number of visits they contributed to the study. Given the distinct patterns of age trajectory between the sexes, we conducted the analysis separately for men and women. Linear mixed-effects growth curve analysis was performed using the *xtmixed* command in Stata 15 (StataCorp LLC, College Station, TX, USA).

The level-one linear mixed-effects growth curve model included the intercept (*β*_0*i*_) and two terms for the age trajectory (linear slope *β*_1*i*_ and quadratic slope *β*_2*i*_):

$${Y}_{ti}={\beta}_{0i}+{\beta}_{1i}{Age}_{ti}+{\beta}_{2i}{Age}_{ti}^2+{e}_{ti}$$

where *Y*_*ti*_ is the weight, height, or BMI value for subject *i* at visit *t.* There were 572,358 participants and the maximal number of visit was 34. *Age*_*ti*_ was the age of participant *i* at visit *t* and is centered at 45 years. *e*_*ti*_ is the within-subject error term (residual).

The level-two linear mixed-effects growth curve model incorporated each participant’s specific characteristics predictors for the age trajectory (as fixed effect) and between-subject variation (as random effects).

For the intercept: *β*_0*i*_ = *γ*_00_ + *γ*_01_*education*_*i*_ + *γ*_02_*cohort*_*i*_ + *u*_0*i*_;

for the linear slope: *β*_1*i*_ = *γ*_10_ + *γ*_11_*education*_*i*_ + *γ*_12_*cohort*_*i*_ + *u*_1*i*_;

for the quadratic slope: *β*_2*i*_ = *γ*_20_ + *γ*_21_*education*_*i*_ + *γ*_22_*cohort*_*i*_ + *u*_2*i*_,

where *γ*_01_, *γ*_11_ and *γ*_21_ represented the effect of education on the intercept and slope and *γ*_02_, *γ*_12_ and *γ*_22_ represented the effect of the birth cohort. Both educational level and birth cohort were coded using dummy variables. *u*_0*i*_, *u*_1*i*_ and *u*_2*i*_ were random effects terms that captured the variation between participants.

To ease the interpretation of the coefficients estimated in the linear mixed-effects growth curve models, we also plotted the predicted mean values for the subjects grouped by sex, birth cohort, and educational levels. These graphs helped us better examine the effect of birth cohort and education on the age trajectory of height, weight, and BMI.

## Results

Table 1 shows the descriptive characteristics of the study sample at baseline. Men accounted for 47.9% (273,879/572358) of the sample. Mean ± standard deviation of age at baseline were 40.1 ± 13.4 years for men and 40.1 ± 13.5 years for women. Most of the study participants were from the 1950–1959 (15.8%), 1960–1969 (24.1%), and 1970–1979 (28.1%) birth cohorts. The distribution of educational levels is also listed in Table 1 by sex, and suggests that men in our sample had higher educational levels than women; 42.9% of men and 32.6% of women attained a university degree or higher. The mean height, weight, and BMI were higher in men (170.0 cm, 69.6 kg and 24.1 kg/m^2^, respectively) than in women (157.5 cm, 55.0 kg and 22.2 kg/m^2^, respectively).

**Table 1:** Baseline characteristics of study sample

Characteristics	Men (*n* = 273,879)	Women (*n* = 298,479)
Age (year), mean ± SD	40.11 ± 13.35	40.09 ± 13.48
Birth cohort (%)		
Before 1920	0.30	0.22
1920–1929	2.62	1.94
1930–1939	6.43	6.56
1940–1949	9.52	11.68
1950–1959	15.23	16.28
1960–1969	25.58	22.65
1970–1979	28.50	27.73
1980–1989	10.85	11.87
After 1990	0.97	1.07
Education (%)		
Some elementary school	1.02	5.89
Elementary school	9.09	14.22
Junior high school	6.30	6.62
Senior high school	20.38	22.26
College	20.32	18.40
University	28.09	25.65
Graduate school or higher	14.80	6.97
Height (cm), mean ± SD	169.96 ± 6.49	157.50 ± 5.88
Weight (kg), mean ± SD	69.56 ± 11.39	54.96 ± 9.08
Body mass index (kg/m^2^), mean ± SD	24.05 ± 3.47	22.19 ± 3.66

*SD* standard deviation

**Fig. 1:**
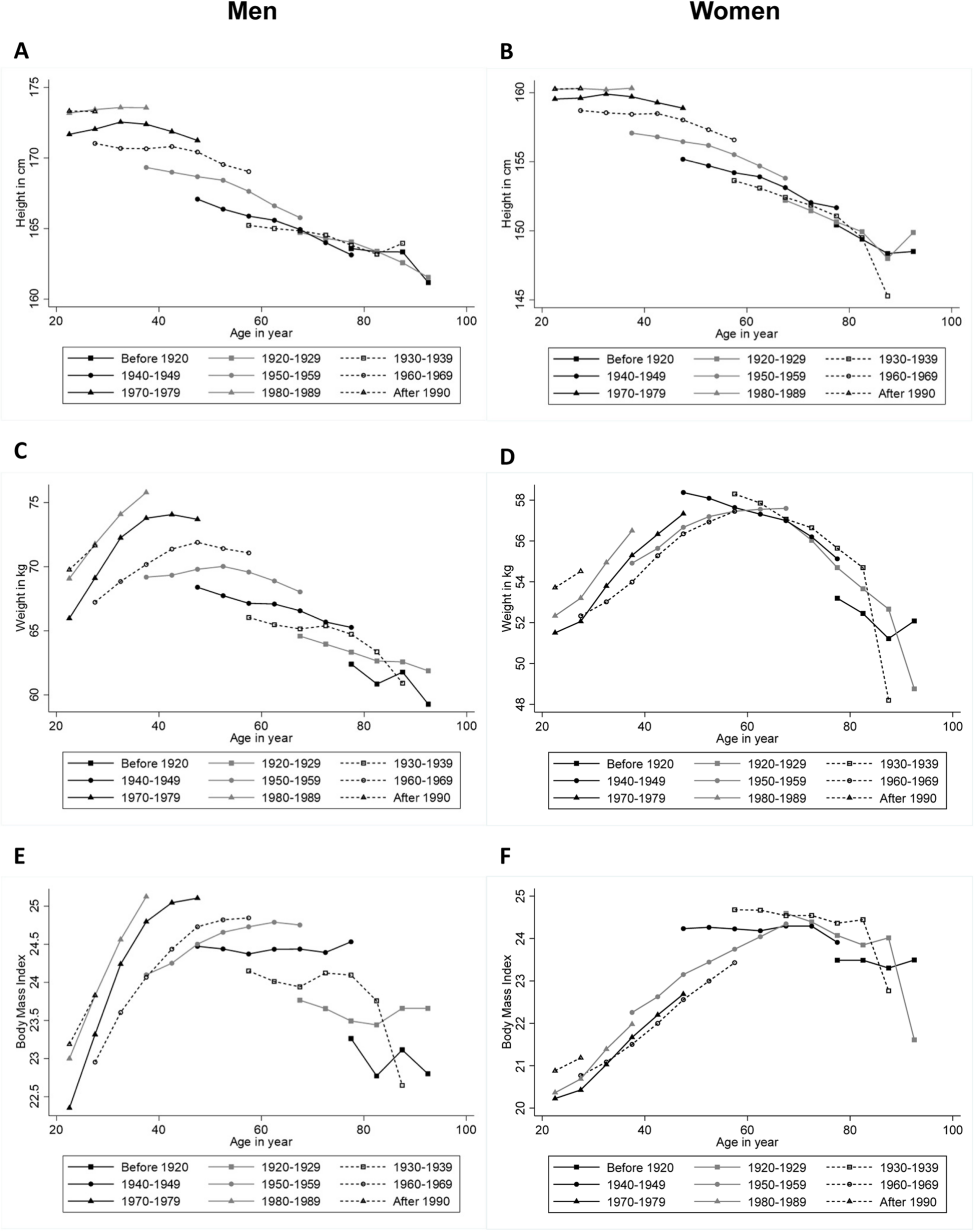
(**A**) Mean height trajectories by birth cohort for men. (**B**) Mean height trajectories by birth cohort for women. (**C**) Mean weight trajectories by birth cohort for men. (**D**) Mean weight trajectories by birth cohort for women. (**E**) Mean BMI trajectories by birth cohort for men. (**F**) Mean BMI trajectories by birth cohort for women. BMI = body mass index

Figure 1 shows the trajectories of mean height, weight and BMI by birth cohorts for men and women. The trajectories of height suggest that as individuals aged in time, the mean height decreased slightly. By comparing different birth cohorts, the younger cohorts had higher mean height values than the older cohorts (see Fig. 1A and B). The trajectories of weight for men suggest that the mean weight increased from young adulthood to about 50 years of age and then declined. Younger birth cohorts had higher mean weight values than older birth cohorts (see Fig. 1C). However, the weight trajectory pattern was different for women. The mean weight for women increased from young adulthood to about age 60 and then declined. The differences in mean weight among birth cohorts were lower for women than for men (see Fig. 1D). The trajectories of BMI for men suggest that the mean BMI increased from young adulthood to about age 50 and then levelled off. Younger birth cohorts also had higher mean BMI values than older birth cohorts (see Fig. 1E). The trajectories of BMI for women increased from young adulthood to about age 70 and then levelled off; the differences in BMI values among birth cohorts for women were not as evident as that for men (see Fig. 1F).

**Fig. 2:**
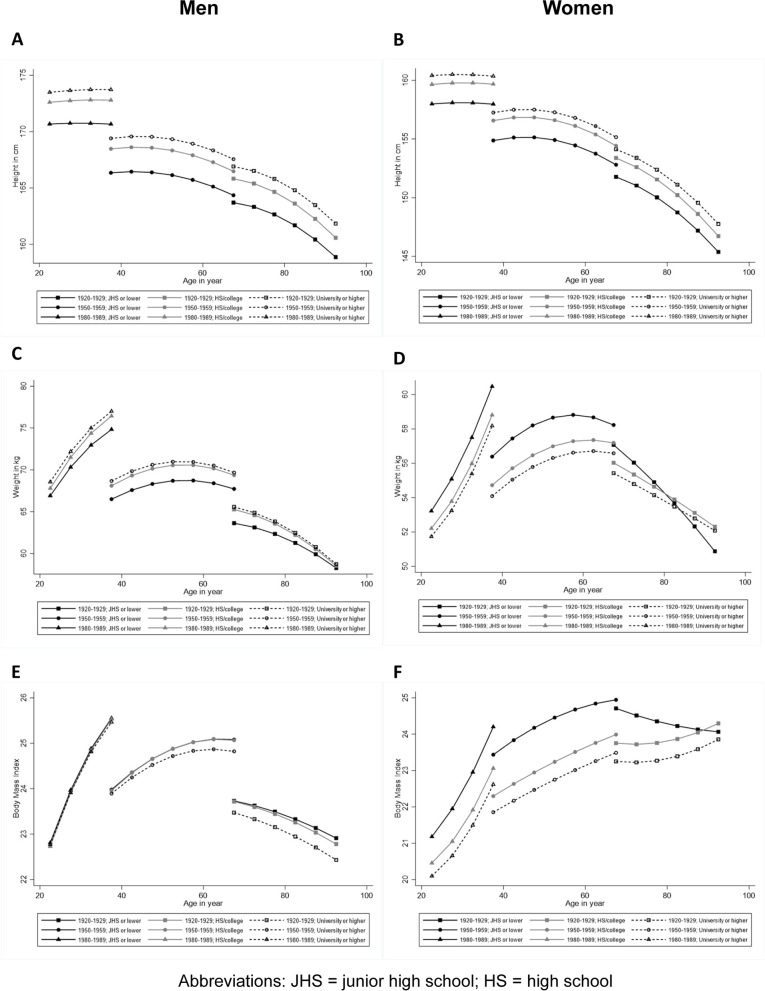
(**A**) Predicted height trajectories by education for men. Only 1920–1929, 1950–1959 and 1980–1989 birth cohorts are included for purpose of illustration. (**B**) Predicted height trajectories by education for women. (**C**) Predicted weight trajectories by education for men. (D) Predicted weight trajectories by education for women. (E) Predicted BMI trajectories by education for men. (F) Predicted BMI trajectories by education for women. BMI = body mass index

Tables 2 and 3 display the coefficients from the linear mixed-effects models of height, weight and BMI among men and women. To ease the interpretation, we used these coefficients to generate graphs of predicted mean trajectories by educational levels (three categories) for men and for women (see Fig. 2). For purposes of illustration, we only graphed the data for birth cohorts of 1920–1929, 1950–1959, and 1980–1989. For height, there were educational disparities for both men and women (see Fig. 2A and B). In the model for men (see Table 2), holding the cohort and age variables constant, men with junior high school or lower education were 3.138 cm (*p* < 0.001) shorter than men with university or higher education. Men with high school/college education were 0.963 cm (*p* < 0.001) shorter than men with university or higher education. In the models for women (see Table 3), women with junior high school or lower education and women with high school/college education were also shorter than women with a university or higher education (effect size: − 2.368, *p* < 0.001 and − 0.664, *p* < 0.001, respectively).

**Table 2:** Linear mixed-effects growth-curve models for men

	Height		Weight		BMI	
	Coefficient	*P*-value	Coefficient	*P*-value	Coefficient	*P*-value
Fixed effect parameters						
For intercept (***β***_**0*i***_)						
Intercept	171.173	< 0.001	72.623	< 0.001	24.767	< 0.001
Education = JHS or lower	−3.138	< 0.001	−2.277	< 0.001	0.121	< 0.001
Education = HS/college	−0.963	< 0.001	− 0.489	< 0.001	0.117	< 0.001
Education = U or higher	Reference					
Cohort = before 1920	−8.143	0.009	−10.806	0.427	−2.448	0.591
Cohort = 1920–1929	−6.275	< 0.001	−7.970	< 0.001	− 1.051	0.047
Cohort = 1930–1939	−4.271	< 0.001	−6.264	< 0.001	−0.966	< 0.001
Cohort = 1940–1949	−3.302	< 0.001	− 4.730	< 0.001	− 0.724	< 0.001
Cohort = 1950–1959	− 1.593	< 0.001	−2.347	< 0.001	− 0.371	< 0.001
Cohort = 1960–1969	Reference					
Cohort = 1970–1979	1.153	< 0.001	2.844	< 0.001	0.646	< 0.001
Cohort = 1980–1989	2.390	< 0.001	5.857	< 0.001	1.219	< 0.001
Cohort = After 1990	6.254	< 0.001	16.950	0.042	4.758	0.088
For linear slope (***β***_**1*i***_)						
Intercept	−0.043	< 0.001	0.134	< 0.001	0.060	< 0.001
Education = JHS or lower	− 0.009	< 0.001	−0.007	0.237	0.005	0.009
Education = HS/college	−0.004	< 0.001	0.010	0.001	0.006	< 0.001
Education = U or higher	Reference					
Cohort = before 1920	0.375	0.019	0.164	0.816	0.003	0.989
Cohort = 1920–1929	0.271	< 0.001	0.056	0.595	−0.056	0.112
Cohort = 1930–1939	0.141	< 0.001	− 0.038	0.274	− 0.060	< 0.001
Cohort = 1940–1949	0.074	< 0.001	−0.024	0.124	−0.033	< 0.001
Cohort = 1950–1959	0.038	< 0.001	0.019	0.002	−0.005	0.017
Cohort = 1960–1969	Reference					
Cohort = 1970–1979	−0.053	< 0.001	−0.194	< 0.001	− 0.047	< 0.001
Cohort = 1980–1989	0.009	0.241	− 0.060	0.147	− 0.027	0.048
Cohort = After 1990	0.322	0.048	0.748	0.326	0.235	0.357
For quadratic slope (***β***_**2*i***_)						
Intercept	−0.004	< 0.001	− 0.012	< 0.001	− 0.003	< 0.001
Education = JHS or lower	0.000	< 0.001	0.001	< 0.001	0.000	0.517
Education = HS/college	0.000	0.273	0.000	0.613	0.000	0.573
Education = U or higher	Reference					
Cohort = before 1920	−0.004	0.082	0.004	0.690	0.001	0.661
Cohort = 1920–1929	−0.002	< 0.001	0.005	0.004	0.002	< 0.001
Cohort = 1930–1939	−0.001	< 0.001	0.008	< 0.001	0.003	< 0.001
Cohort = 1940–1949	0.000	0.970	0.007	< 0.001	0.002	< 0.001
Cohort = 1950–1959	0.000	0.556	0.004	< 0.001	0.001	< 0.001
Cohort = 1960–1969	Reference					
Cohort = 1970–1979	− 0.001	< 0.001	− 0.011	< 0.001	− 0.003	< 0.001
Cohort = 1980–1989	0.002	< 0.001	−0.005	< 0.001	− 0.002	< 0.001
Cohort = After 1990	0.008	0.024	0.012	0.478	0.004	0.544
Random effects parameters						
Intercept (***u***_**0*i***_)	33.597		120.480		11.174	
Linear slope (***u***_**1*i***_)	0.002		0.129		0.015	
Quadratic slope (***u***_**2*i***_)	0.000		0.000		0.000	
Residual (***e***_***ti***_)	0.213		4.312		0.504	

*JHS* junior high school; *HS* high school; *U* University

**Table 3:** Linear mixed-effects growth-curve models for women

	Height		Weight		BMI	
	Coefficient	*P*-value	Coefficient	*P*-value	Coefficient	*P*-value
Fixed effect parameters						
For intercept (***β***_**0*i***_)						
Intercept	158.875	< 0.001	55.764	< 0.001	22.092	< 0.001
Education = JHS or lower	−2.368	< 0.001	2.417	< 0.001	1.691	< 0.001
Education = HS/college	−0.664	< 0.001	0.672	< 0.001	0.471	< 0.001
Education = U or higher	Reference					
Cohort = before 1920	−1.182	< 0.001	−1.061	0.414	−0.251	0.628
Cohort = 1920–1929	−4.743	< 0.001	2.234	0.222	2.201	0.003
Cohort = 1930–1939	−3.947	< 0.001	0.158	0.631	1.195	< 0.001
Cohort = 1940–1949	−2.709	< 0.001	0.381	< 0.001	0.927	< 0.001
Cohort = 1950–1959	−1.340	< 0.001	− 0.318	< 0.001	0.229	< 0.001
Cohort = 1960–1969	Reference					
Cohort = 1970–1979	0.757	< 0.001	2.533	< 0.001	0.797	< 0.001
Cohort = 1980–1989	1.100	< 0.001	7.791	< 0.001	2.734	< 0.001
Cohort = After 1990	7.892	< 0.001	24.465	0.001	7.895	0.006
For linear slope (***β***_**1*i***_)						
Intercept	−0.045	< 0.001	0.234	< 0.001	0.108	< 0.001
Education = JHS or lower	0.002	0.203	0.003	0.472	0.008	< 0.001
Education = HS/college	0.000	0.639	0.003	0.382	0.003	0.019
Education = U or higher	Reference					
Cohort = before 1920	0.295	< 0.001	−0.086	0.307	−0.090	0.007
Cohort = 1920–1929	0.165	< 0.001	−0.339	0.006	−0.187	< 0.001
Cohort = 1930–1939	0.148	< 0.001	−0.217	< 0.001	− 0.135	< 0.001
Cohort = 1940–1949	0.096	< 0.001	−0.238	< 0.001	− 0.131	< 0.001
Cohort = 1950–1959	0.046	< 0.001	− 0.086	< 0.001	−0.048	< 0.001
Cohort = 1960–1969	Reference					
Cohort = 1970–1979	−0.045	< 0.001	0.150	< 0.001	0.071	< 0.001
Cohort = 1980–1989	−0.022	0.005	0.579	< 0.001	0.229	< 0.001
Cohort = After 1990	0.557	0.001	1.835	0.006	0.611	0.020
For quadratic slope (***β***_**2*i***_)						
Intercept	−0.004	< 0.001	− 0.002	< 0.001	0.000	< 0.001
Education = JHS or lower	0.000	0.524	−0.002	< 0.001	−0.001	< 0.001
Education = HS/college	0.000	< 0.001	0.000	0.138	0.000	0.260
Education = U or higher	Reference					
Cohort = before 1920	−0.006	< 0.001	−0.004	0.032	0.000	0.728
Cohort = 1920–1929	−0.002	0.002	0.002	0.471	0.001	0.192
Cohort = 1930–1939	−0.002	< 0.001	0.001	0.241	0.001	0.003
Cohort = 1940–1949	−0.001	< 0.001	0.002	< 0.001	0.001	< 0.001
Cohort = 1950–1959	−0.001	< 0.001	−0.002	< 0.001	− 0.001	< 0.001
Cohort = 1960–1969	Reference					
Cohort = 1970–1979	0.000	0.005	0.003	< 0.001	0.001	< 0.001
Cohort = 1980–1989	0.002	< 0.001	0.015	< 0.001	0.005	< 0.001
Cohort = After 1990	0.014	< 0.001	0.041	0.007	0.013	0.026
Random effects parameters						
Intercept (***u***_**0*i***_)	27.765		82.198		11.549	
Linear slope (***u***_**1*i***_)	0.002		0.083		0.013	
Quadratic slope (***u***_**2*i***_)	0.000		0.000		0.000	
Residual (***e***_***ti***_)	0.218		3.711		0.601	

*JHS* junior high school; *HS* high school; *U* University

For weight, Fig. 2C and D also suggest educational disparities, but the direction was reverse when comparing men versus women. In the model for men (see Table 2), men with junior high school or lower education and men with high school/college education were lighter than men with university or higher education (effect size: − 2.277, *p* < 0.001 and − 0.489, *p* < 0.001, respectively). But in the model for women (see Table 3), women with junior high school or lower education and women with high school/college education were heavier than women with university or higher education (effect size: 2.417, *p* < 0.001 and 0.672, *p* < 0.001, respectively). We found that women with junior high school or lower education had a faster decline in weight after age 70.

For BMI, the educational disparities were greater in women than in men (see Fig. 2E and F). Men with junior high school or lower education and men with high school/college education had higher BMI values than men with university or higher education (see Table 2, effect size: 0.121, *p* < 0.001 and 0.117, *p* < 0.001, respectively). Women with junior high school or lower education and women with high school/college education also had higher BMI values than women with university or higher education (see Table 3, effect size: 1.691, *p* < 0.001 and 0.471, *p* < 0.001, respectively).

## Discussion

Our study had some noteworthy findings. First, the age-related patterns for the trajectories of weight and BMI were shown and differed between men and women. The mean weight and BMI increased and peaked at an older age in women (60–70 years) than in men (40–50 years). Second, there was evidence of cohort effects on the trajectories of height, weight, and BMI in the Taiwanese adults born from 1920 to 1990. Over secular time, the mean height, weight, and BMI had increased, and the cohort effects were more evident for men than for women. Third, education had distinct influences on the trajectories of weight and BMI. Lower educational levels were associated with lower weight in men but with higher weight in women. For both men and women, lower educational levels were associated with higher BMI and the disparities were found to be larger among women.

The age-related pattern for the trajectory of BMI increased from 20 to 50 years of age in men, but the pattern in women increased from 20 to 70 years of age. Women continued to have increasing BMI after age 50, perhaps due to the effect of menopause transition. During menopause transition, most women experience the physiological changes and their function of ovaries is declining, resulting in the unpredictable fluctuation of sex hormones (e.g., estrogen and progesterone). Sex hormones such as estrogen seem to have a cardio-protective effect on women, leading to reduced oxidative stress and fibrosis, and to increased angiogenesis and vasodilation [19]. Several longitudinal studies have also shown that menopause transition is related to increases in waist circumference, metabolic syndrome, and cholesterol levels [20–22]. It is important to study and understand the influence of menopause transition on the body composition and cardio-metabolic health, as this is a universal and crucial stage in the life course for all women.

We observed evident cohort effects on the trajectories of height, weight, and BMI; younger cohorts had higher values than the older cohorts. These results indicate that the historical changes in the physical and social environment might have a strong effect on body type and composition. In the older birth cohorts in Taiwan, there was a larger proportion of people who suffered from poverty and undernutrition. Since the industrialization and economic growth of Taiwan, the socioeconomic condition and nutritional status has become better and better, which has resulted in the increasing height and weight over secular time. In the youngest birth cohorts, there was probably the highest proportion of people having unhealthy eating behaviors, sedentary lifestyles, and using technologies that are conductive to obesity. Our observations regarding the cohort effects agree with previous findings from other countries and highlight that the social and physical environments that individuals are exposed to play an important role in the trajectory of BMI, in addition to biological aging [5, 6, 8, 23, 24].

Furthermore, we observed a larger cohort effect for men than for women in our study population. In a study using the China Health and Nutritional Survey, the investigators showed similar findings [5]. In their final adjusted linear mixed-effects model, comparing an average 25- or 45-year-old person in 2009 versus 1991 in China, they found that the change in BMI was greater for men than for women. The authors hypothesized that occupation may cause the sex differences. More and more people have been engaged in occupations with lower levels of physical activity than before and the tendency has been stronger for men than for women [25, 26]. We think that similar trends could be observed in Taiwan, and the findings also imply that occupational physical activity may account for a major part of total energy expenditures, particularly for men [24, 27].

We also identified a clear “social patterning” of the trajectories of BMI in our study population. Individuals with higher educational levels may have better health literacy, socio-psychological resources, and self-efficacy, and be more likely to engage in healthy behaviors to control their weight gain [28]. Thereby, we found higher educational levels were associated with lower weight and BMI among women. However, for men the educational disparity of BMI was smaller than for women and higher educational levels were even associated with higher weight among men. Similar findings were reported in other populations. In the US, Yang and colleagues [8] reported a clear educational gradient in the trajectories of BMI for women and showed that women with a college degree or more had the lowest BMI trajectory, but this gradient was less evident for men. In a comparative study between Japan and England [29], Martikainen and colleagues found that Japanese men with higher employment grades and higher educational levels had higher BMI, waist-to-hip-ratio and lower high density lipoprotein cholesterol, whereas in England higher socioeconomic status had better profiles regarding these risk factors. The social patterning observed in our population may suggest that education is a more important social determinant for cardio-metabolic health for women than for men and that there is variation among different countries and cultures in terms of educational disparities.

The major strength of the current study was that weight and height were measured objectively rather than relying on self-reporting of the participants. Many reports have collected only the self-reported data on weight and height. People tend to overreport their height and underreport their weight [30]. Another strength is that our open cohort design and study sample comprised 572,358 individuals aged between 20 and 94 years who were followed up to 22 years, which provided a unique opportunity for doing life course trajectory research as the sample included people from a wide spectrum of birth cohorts.

Nevertheless, our study was not free of limitations. People with a normal BMI may be overrepresented in the older participants of our sample, since people who were underweight, overweight or obese tended to have a higher risk of death [1]. Thereby, the trajectories of height, weight, and BMI might be subject to this selective mortality in our study. Another limitation was that we did not specifically add the period effect into our models given the model identification problems of the collinearity of age, period, and cohort effects [31]. As in the study done by Yang et al. [8], we assumed that the period effect was trivial and that we would detect the period effect through the cohort-by-age interaction terms.

## Conclusion

Trends of increasing BMI are likely to result in subsequent increases in the prevalence of overweight and obesity in the population. Our study demonstrated that in Taiwan there is a birth cohort effect on the increasing BMI, particularly for men, and that there is an educational disparity for trajectories of BMI, particularly for women. These findings provide important insights to the design and implementation of interventions that address overweight and obesity; for example, we should put more emphasis on the younger cohorts at an early age and on women with lower educational levels.

## Data Availability

The datasets used and analyzed during the current study are available from the corresponding author (Prof. Tsung Yu) on reasonable request.

## Abbreviations

BMI: Body mass index
HS: High school
JHS: Junior high school
SD: Standard deviation
US: United States
